# Modeling antibiotic resistance in the microbiota using multi-level Petri Nets

**DOI:** 10.1186/s12918-018-0627-1

**Published:** 2018-11-22

**Authors:** Roberta Bardini, Stefano Di Carlo, Gianfranco Politano, Alfredo Benso

**Affiliations:** Politecnico di Torino, Control and Computer Engineering Department, Corso Duca degli Abruzzi 24, Torino, 10129 Italy

**Keywords:** Antibiotic resistance, Human microbiota, Hybrid models, Computational systems biology, Petri Nets, Nets-Within-Nets

## Abstract

**Background:**

The unregulated use of antibiotics not only in clinical practice but also in farm animals breeding is causing a unprecedented growth of antibiotic resistant bacterial strains. This problem can be analyzed at different levels, from the antibiotic resistance spreading dynamics at the host population level down to the molecular mechanisms at the bacteria level. In fact, antibiotic administration policies and practices affect the societal system where individuals developing resistance interact with each other and with the environment. Each individual can be seen as a meta-organism together with its associated microbiota, which proves to have a prominent role in the resistance spreading dynamics. Eventually, in each microbiota, bacterial population dynamics and vertical or horizontal gene transfer events activate cellular and molecular mechanisms for resistance spreading that can also be possible targets for its prevention.

**Results:**

In this work we show how to use the Nets-Within-Nets formalism to model the dynamics between different antibiotic administration protocols and antibiotic resistance, both at the individuals population and at the single microbiota level. Three application examples are presented to show the flexibility of this approach in integrating heterogeneous information in the same model, a fundamental property when creating computational models complex biological systems. Simulations allow to explicitly take into account timing and stochastic events.

**Conclusions:**

This work demonstrates how the NWN formalism can be used to efficiently model antibiotic resistance population dynamics at different levels of detail. The proposed modeling approach not only provides a valuable tool for investigating causal, quantitative relations between different events and mechanisms, but can be also used as a valid support for decision making processes and protocol development.

## Background

Humans, like other multicellular organisms, can be considered as meta-organisms composed of a host and its symbiotic commensal microbiota adapted to the niches the host provides as living environment. The human microbiota, i.e., the microbial taxa associated with humans, has an estimated population of a hundred trillion bacterial symbionts, which outnumber the host cells by at least a factor of ten, and express at least ten fold more unique genes than their host’s genome [1]. The catalog of these organisms and their genes represents the human microbiome [2, 3]. Each microbiota corresponds to different coexistent species, which altogether live in a symbiotic relationship with the human body: microorganisms obtain to live in a favorable niche, while the host gets different sorts of advantages [4]. In the frame of a symbiotic relationship with the host, bacteria and other organisms composing the microbiota normally do not express virulent traits. Indeed, one of their functions is to protect the host from pathogens [5].

These complex communities of microbes that include bacteria, fungi, viruses and other microbial and eukaryotic species, provide a tremendous enzymatic capability and play a fundamental role in controlling several aspects of the host’s physiology. For example, the gut microbiome, which is by far the most extensively studied, largely contributes to processing nutrients in the gastrointestinal tract, affecting in a significant way the composition of what the body actually absorbs from the nutrients in transit [6]. Different gut microbiomes can extract different amounts of energy from the same nutrients, as shown in [7]. Over the past few years, the field of immunology has been revolutionized by the growing understanding of how the microbiota is able to affect the induction and education of the mammalian immune system [8]. This trend in the research is supported by a steadily increasing number of launched worldwide projects on the topic [9, 10].

Different bacterial species are present in different quantities depending on the specific microbiota. This depends on how much their growth dynamics, which are constrained by the overall availability of resources, let them conquer the ecological space [11]. The more space a bacterial species takes, the more its genetic set affects the overall functional set of the microbiota. But the dynamic functional profile of a microbiota takes shape after other factors as well.

Healthy microbiotas share two common features: (i) a uniform, trans-species core of functionalities in the corresponding microbiomes, and (ii) a great diversity in terms of species composition. This provides on one side a set of guaranteed core functionalities and, on the other side, a great capability for plasticity [2]. The microbiota can recruit new functionalities in two ways:

1. new species can join the population bringing in their different functional sets and then enlarging the functional capabilities of the pre-existing microbiota [12];
2. microbic cells can activate different *Horizontal Gene Transfer* (HGT) mechanisms, causing the acquisition of new functional capabilities [13]. The most studied HGT mechanism with respect to the spreading of antibiotic resistance is the exchange of plasmids between bacterial cells from the same or from different species [14].

In this work, we show how to model antibiotic resistance among bacterial cells living in human microbiotas. Such phenomenon proves to be promoted by the administration of drugs originally intended to prevent or cure infections caused by pathogenic microbes. Antimicrobials are a class of drugs targeting pathogenic traits in diverse microbial species, ranging from viruses to parasites, while antibiotics target more specifically bacterial species as pathogens. Both classes of drugs can cause insurgence and spread of resistance in all the bacterial populations they come in contact with.

Since most antibiotics tend to target bacteria through very conserved biological features [15], most of the times they hit a large spectrum of different bacterial species at the same time. After antibiotic administration, many bacterial cells (besides the pathogenic species the aggression was directed onto) perish. Only those expressing antibiotic resistance towards that antibiotic survive. The death of non-resistant bacterial populations frees a large portion of the ecological space: resistant bacteria have way less competition for resources, and thus a great advantage in colonizing the niche [16]. When colonizing new ecosystems, HGT plays a central role in the acquisition of new capabilities, antibiotic resistance included. The different HGT mechanisms involve diverse genetic material, corresponding to a wide range of different functionalities. Most times, plasmid-mediated HGT mechanisms prove to be the most relevant for the spread of antimicrobial and antibiotic resistance within a microbiota [17]. Such events are part of the physiology of interactions among bacterial cells, providing a substrate for the microbiota’s plasticity and adaptability to a changing environment. Among the functionalities that bacterial cells can transfer through HGT, the acquisition of antibiotic resistance is a good example of adaptation to an hostile environment: antibiotic treatment is an environmental threat to them, and resistance provides an evolutionary advantage. Exchanges of genetic information through colonization by new species or HGT mechanisms can take place not only within a microbiota, but between different microbiotas as well, thanks to the contacts its host has with the environment and other hosts. Still, the dynamics underling the spread are complex, and involve mechanisms related to the bacterial cells and populations, as well as to the host organisms, and their contacts with the environment, including the different types of antibiotic administration protocols that can be implemented.

Antibiotic resistance is therefore not only a problem of the individual host, but it rather affects the overall system in which the hosts live. The spread of antibiotic resistance can definitely be framed in terms of imminent risk for the society, for the production system, as well as for the environment in general [18].

In this work, we introduce a computational tool to investigate how different antibiotic protocols can affect the spread of antibiotic resistance at different levels: from health care management of antibiotic treatment policies and practices, to individual patients as meta-organisms carrying peculiar and evolving microbiotas. In other words, we tackle this problem under a systems biology perspective, creating a computational model that properly integrates and manages information from the different system levels of interest. This work extends the preliminary results previously published in [19] by the same authors.

The proposed model is based on Nets-Within-Nets (NWN), a high-level Petri Nets formalism supporting the development of multi-level and hybrid models suitable for stochastic and timed dynamic simulations [20, 21].

NWN models are powerful tools to represent hierarchy and encapsulation of a system, two important characteristics of the biological complexity. Moreover, they support the use of different degrees of abstraction for different parts of the same model. In fact, when modeling a multi-level biological system, the availability of information is often not uniform for all portions of the system, or information about different system elements or levels is specified under different formalisms, each one having different ways of extracting information from data. Also, as a modeling strategy, the modeler could choose to use the formalism differently for different parts of the model. This can be done with the aim of finding a good trade-off between complexity of the model and accuracy of the representation.

In general, intended in these ways, the flexibility of the NWN formalism allows to integrate into a single consistent model several heterogeneous information, enabling to derive simulation outcomes and predictions out of a wide part of the biological complexity intrinsic to the system [20]. In some cases, this can result in higher accuracy compared to models addressing a single system level.

All these characteristics make NWN models a good option to build decision support tools for the simulation and analysis of antibiotic resistance spreading in selected contexts. The possibility of simulating the evolution of the whole system based on different starting conditions and external stimuli is a powerful tool to support the design, optimization and testing of innovative therapeutic protocols and for policy making in the health care context.

## Methods

This section overviews the proposed computational model, which is able to simulate how different antibiotic protocols can affect antibiotic resistance at the microbiota level. After introducing the NWN formalism and motivating its use for our modeling goals, a set of use cases is described, introducing the related experimental designs. The capabilities of the proposed modeling approach must be considered both as a tool to produce new insights on biological systems interested by the spread antibiotic resistance, and as a tool for supporting decision making processes.

### Nets-Within-Nets

Our modeling strategy is based on the Nets-Within-Nets formalism, a high-level Petri Nets formalism supporting multi-level and hybrid model specifications as well as stochastic dynamic simulations [22].

In order to better explain the advantages provided by the NWN when modeling biological systems, we start with a brief recapitulation of the basic concepts and definitions behind the Petri Nets formalism, and its incrementally complex extensions.

As reviewed in [23], low-level Petri Nets are a valuable state-of-the-art tool for computational biology. They combine usability and simplicity in model design with the capability of supporting dynamic simulations and formal, quantitative analysis. Petri Nets at their core are bipartite graphs with two kinds of nodes: places and transitions. Each place can contain a number of tokens, which are also elements of the formalism providing a quantitative and discrete representation of resources. Each transition has rules regulating its enabling and activation. Directed arcs link places and transitions to form the desired network architectures. This supports the modeling and simulation of distributed systems and processes running in parallel and competing for resources (i.e., tokens), a typical characteristic of several biological system. More specifically, low-level Petri Nets suit the modeling requirements of biological processes considering a single organizational system level, and a single or few types of resources. For example, Petri Nets can be exploited to model a metabolic network. Places can be used to represent the molecular species involved in relevant biochemical reactions and the enzymes carrying out that reactions. Tokens can be used to represent discrete quantities of such biomolecules. Eventually, transitions can be used to model the stoichiometric rules and kinetics of reactions.

The modeling capabilities of low-level Petri Nets are limited when trying to account in a quantitative and holistic way for the biological complexity of larger and more complex systems. High-level formalisms extend low-level Petri Nets, making it possible to represent more of the system’s complexity. For example, in Colored Petri Nets each token can carry structured information, allowing to model different types of resources. Stochastic Petri Nets support stochastic behaviors in simulations, while Timed Petri Nets allow for the setting of specific timings for transition activations. Among other high-level formalisms, the NWN formalism provides the capability of expressing a fundamental feature of biological systems: the hierarchical organization of multiple system levels encompassing a range of time and space scales [24]. Moreover, a NWN follows an object-oriented paradigm expressing encapsulation and selective communication, making it possible to suitably represent biological compartmentalization and semi-permeability of biological membranes. To allow a hierarchical organization, tokens under the NWN formalism can be specified and simulated using the same NWN formalism [22] and are therefore called *net-tokens*. They are therefore instances of other NWN models, living within and being simulated concurrently with a higher-level network instance. In addition to such nested architectures, NWN models may include extensions from other high-level formalisms, such as stochastic rules, colored tokens and timings. In particular, the capability of managing colored tokens, coupled with the hierarchical organization makes it easy to handle in a consistent way different information structures in the same model. This is a valuable tool when building complex biological models since different organizational levels of the model may have different ways of extracting data from experiments and information form data (interested readers may refer to [21] for a thorough analysis of these aspects).

In our opinion, for all these reasons, NWN models fulfill the modeling requirements posed by complex biological systems and by the diverse contributors to the the relative knowledge generation process. This holds for the problem considered in this paper, where different system parts evolve in a mostly independent way, at different organizational levels, exchanging information in a highly regulated and selective way.

For model design and simulation we rely on Renew, an integrated tool supporting the design and simulation of high-level Petri Nets [25]. Renew follows the object-oriented programming paradigm in which net-tokens are instances of net classes encapsulated into higher level nets. Renew is based on Java: each net is in fact a Java class that can in turn be interfaced with any other class and standard library, thus enabling to tailor the models to the specific design needs [25]. For the sake of completeness all models presented in this paper are reported using the full Renew syntax, thus allowing readers to easily reproduce the model in their computational environments. To help readers to familiarize with this syntax, annotation through colors and labels has been used in the figures to highlight the high-level concepts of the proposed models.

### Model construction

The main goal of this paper is to show how the NWN formalism can be used to address the question of how antibiotic treatment protocol design and strategies relate to the insurgence, spread and severity of antibiotic resistance within both individual human microbiotas and populations of human hosts [12].

To demonstrate the NWN flexibility and performances, we start from the presentation of an abstract model (hereinafter referred to as *General model*) introducing the most important actors involved in antibiotic resistance and their main interactions. Being abstract, this model is analyzed resorting to generic parameters that do not have a direct relation with a specific biological setup but recapitulate the antibiotic resistance mechanism at a qualitative level [26]. To show the proposed model at work, the second model customizes the general framework to analyze the specific case of antibiotic resistance in the mouse gut microbiota considering the contribution of bacterial predation mechanisms involving Acinetobacterium and E. Coli bacterial cells [27, 28]. In this specific case, realistic parameters (e.g., bacterial populations size, HGT rate, etc.) are identified from the literature and plugged into the proposed model as will be described in the following sections.

In both cases the goal of the proposed model is to simulate and observe to what extent mild or severe resistance spread within a single microbiota and in a population of hosts. This is evaluated comparing: (1) a control population not receiving any treatment, (2) a population receiving traditional antibiotic treatment, and (3) a population receiving antibiotics under a carefully designed innovative administration protocol.

Figure 1 shows a high-level conceptual view of the two models, organized into three hierarchical levels:

1. the Hosts (top level),

**Fig. 1:**
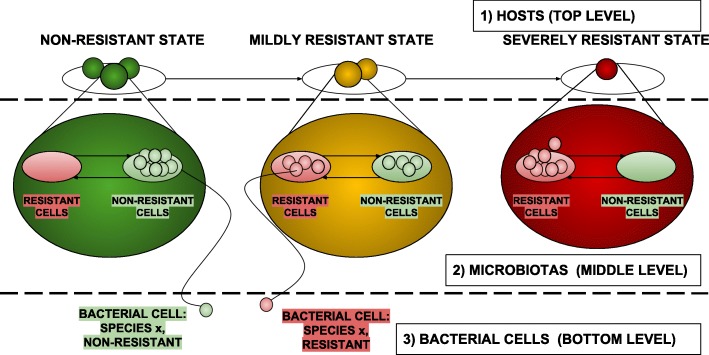
High level conceptual view of the proposed computational model organized into three hierarchical levels
2. the Microbiotas (middle level), and
3. the Bacterial Cells (the bottom level).

#### General model

The first proposed model is a general representation of the most important actors involved in antibiotic resistance.

#### Host level (top level)

**Fig. 2:**
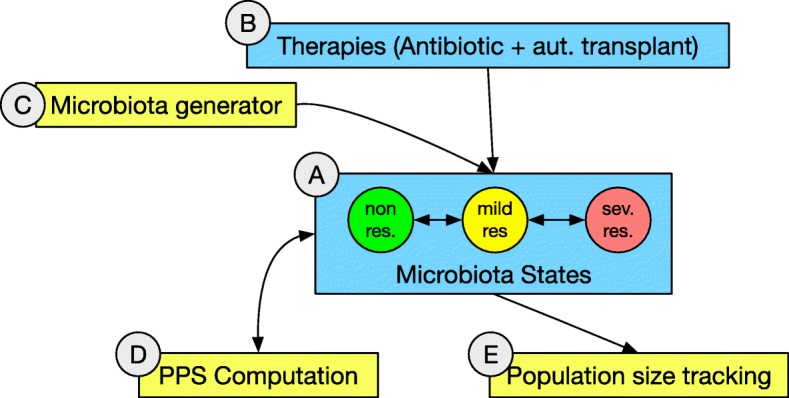
Conceptual view of the network architecture for the hosts (top) level. Three main places describe the health states the microbiotas can assume. The letters identify the different sections of the detailed model presented in Fig. 3

**Fig. 3:**
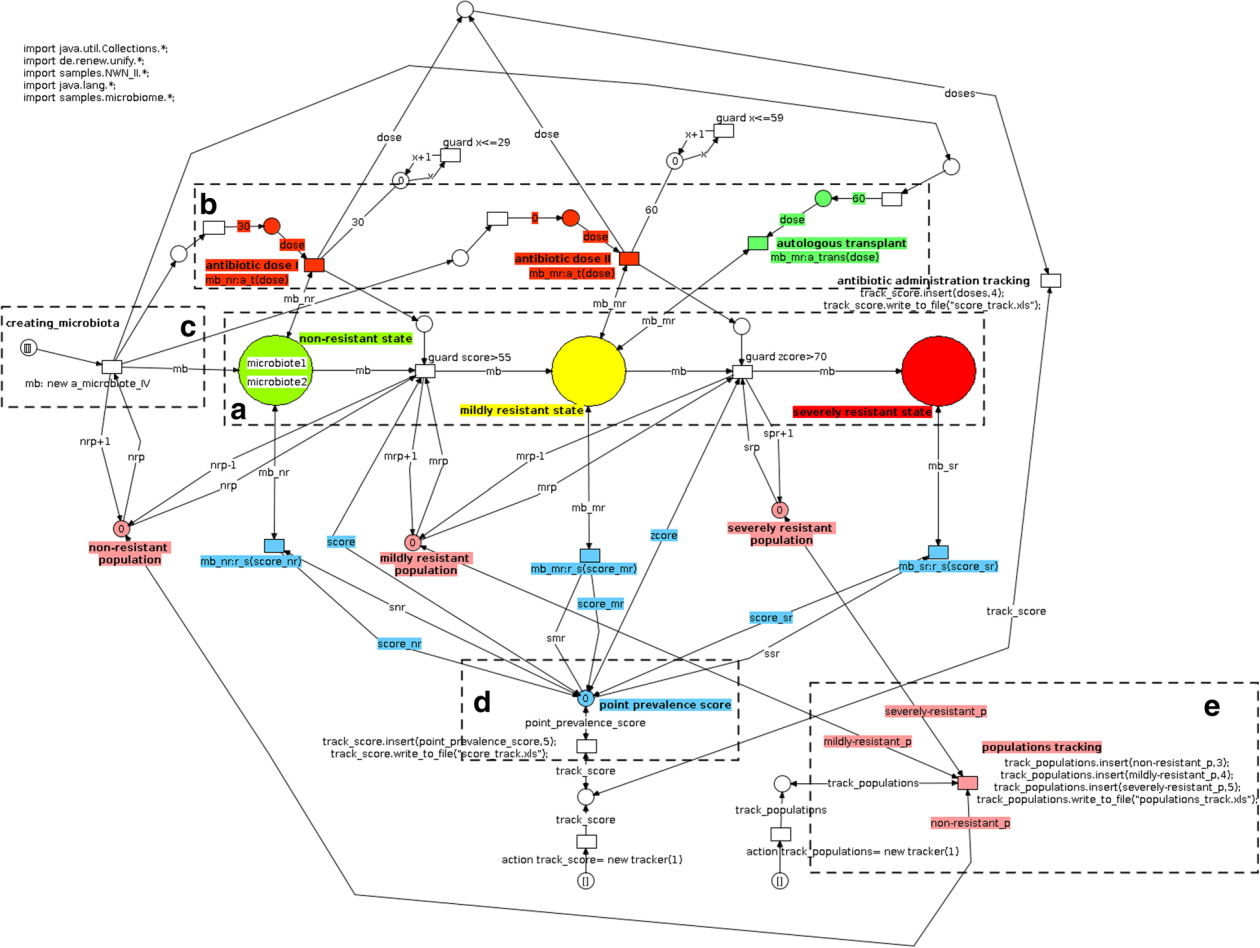
Network architecture for the hosts (top) level. Three main places describe the health states the microbiotas can assume: the non-resistant (in green), mildly resistant (in yellow) and severely resistant (in red) states respectively (**a**). Transitions can move microbiota tokens, each having the network structure from Fig. 4, to the next place. The state of non-resistance holds two microbiota tokens depicted in a compact form, i.e., with their name only. This happens according to the value of their point prevalence score which a synchronous channel (**d**) reads from networks at the lower level, possibly taking the relative microbiota to the next step along resistance progression (the structures in **e** track the changing numerosity of microbiota instances in each place). Synchronous channels take care of the antibiotic administration and microbiota integration events (**b**), activating network structures at the lower level (Fig. 4b and f, respectively) according to time delays and number of microbiota instances injected in the network by the dedicated structure (**c**)

This level, whose complete model is reported in Figs. 2 and 3, describes the population of individuals in which antibiotics resistance can spread. Figure 2 summarizes the main functional blocks that make the model; blue boxes represent biological sub-systems, whereas yellow boxes are input or observation blocks. Figure 3 shows the actual Petri-Net implementation of the Host level.

**Fig. 4:**
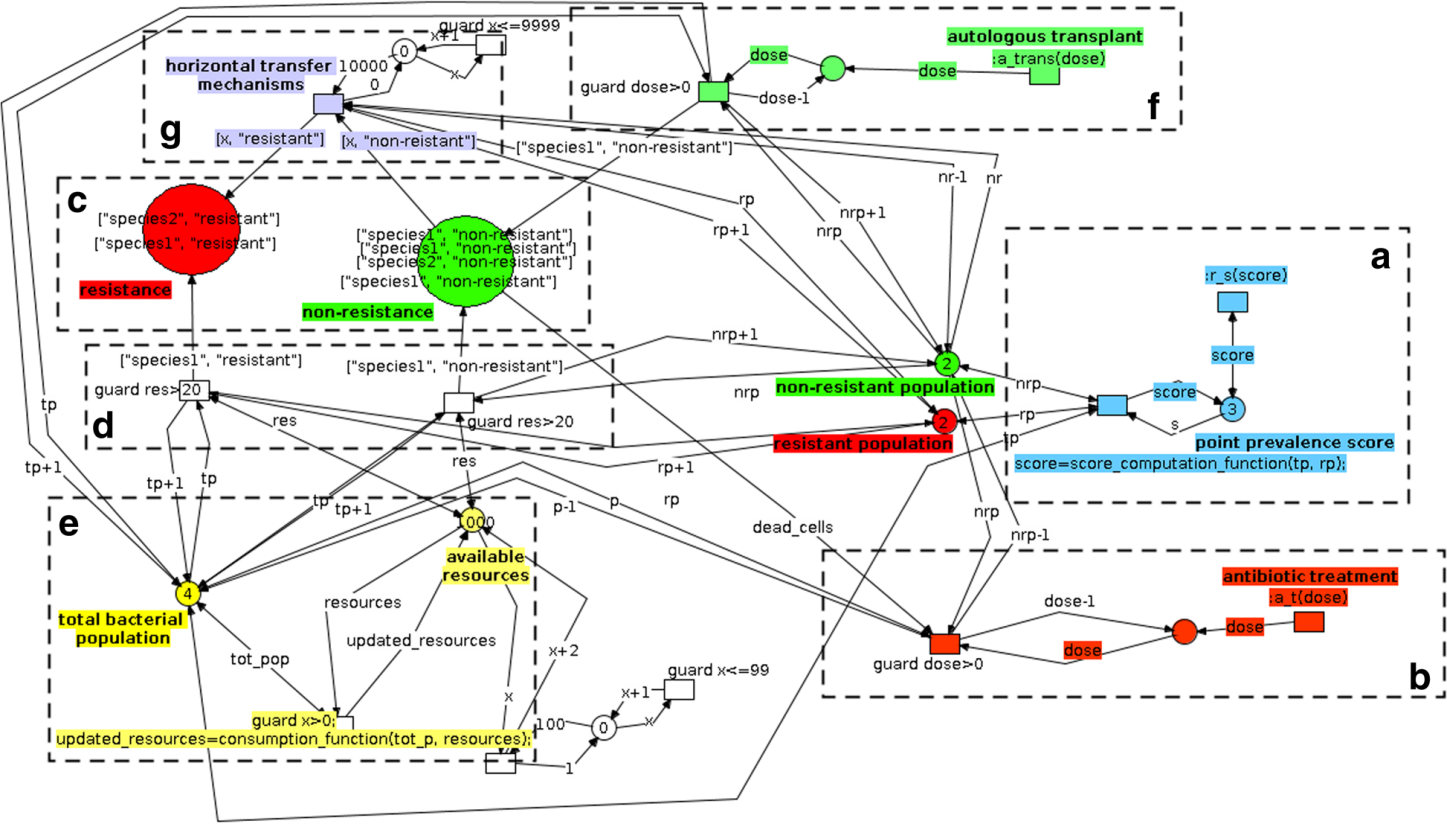
Network architecture for the microbiotas (median) level. Two main places describe two conditions each bacterial cell can assume: a state of non-resistance (green) and a state of resistance (red), respectively (**c**). Horizontal transfer mechanisms can turn non-resistant cells into resistant ones (**g**). Network structures managing the generation of new bacterial cells (**d**), total population numerosity and resources availability (**e**) give rise to a competitive population dynamics between resistant and non-resistant populations. Dose-dependent depletion of non-resistant cells following antibiotic administration (**b**) and microbiota reintegration (**f**) events activate as synchronous channels with structures in Fig. 3b. The structure in **a** dynamically computes the point prevalence score of the microbiota network, making the information available for the upper level through a synchronous channel (see Fig. 3d)

At this level three places are dedicated to model the possible conditions a microbiota assume with respect to antibiotic resistance capabilities (as in Fig. 3a): (i) the place in green refers to a condition of absence of resistance, (ii) the place in yellow models a state of mild resistance, and (iii) finally the place in red reports a state of severe resistance. Transitions between such places represent the steps towards progressive worsening of the antibiotic resistance state of the microbiota they are moving. At this level, each net-token represents an individual or, more specifically, the microbiota of an individual. Each net-token/microbiota is therefore associated to an instance of a net class whose structure is reported in Fig. 4. At the Host level, each token/microbiota is evaluated by means of its *point prevalence score* (PPS), that is dynamically computed during the simulation as the proportion of bacterial cells carrying resistance factors over the total bacterial population [29] (Fig. 4a); the PPS value is used at the upper network-level to take decisions concerning whether the net-token should or shouldn’t be moved to the next place (Fig. 3d). More specifically, if the PPS exceeds the threshold set as the upper bound for the current state resistance, transitions move the microbiota net-token to the next stage of resistance progression, i.e., to the next place at the host level (Fig. 3a). In this model, PPS variations arise primarily from antibiotic administration and microbiota preventive re-integration (say, autologous transplants) events, modeled by means of activation of administration mechanisms at the host and microbiota levels (Figs. 3b; 4b and 4f). These model structures are designed to affect both the population level, where microbiotas exist as net-tokens (Fig. 3c presents the network structure producing instances of them) and at the level of individual microbiotas (Fig. 4). A backend of custom Java classes tracks the point prevalence scores (Fig. 4d) as well as the the number of individuals in the three different states (Fig. 4e).

#### Microbiota level (median level)

**Fig. 5:**
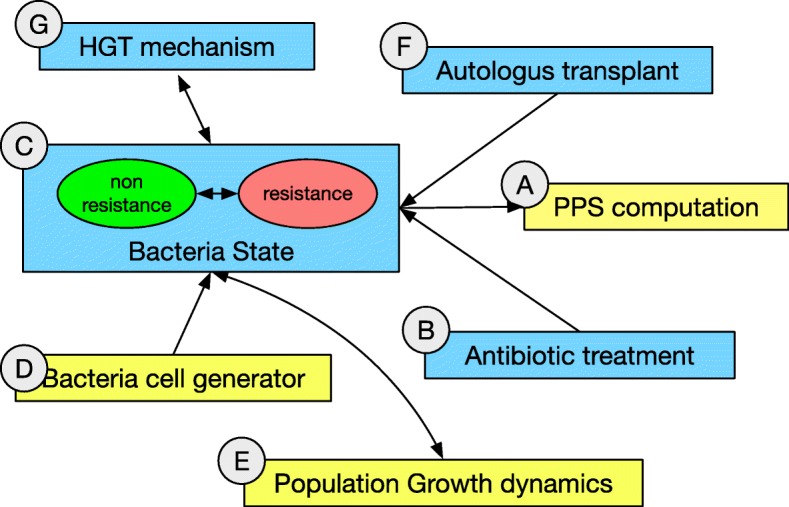
Conceptual view of the network architecture for the microbiotas (median) level. Two main places describe two conditions each bacterial cell can assume. The letters identify the different sections of the detailed model presented in Fig. 4

Each microbiota exists at the hosts (top) level as a net-token associated with an instance of the microbiota network depicted in Figs. 5 and 4. As in the case of the top-level, Fig. 5 reports a high-level view of the network while Fig. 4 shows the detailed implementation.

In this more general model, at the microbiota level two places (Fig. 4c) host tokens representing bacterial cells: red tokens represent bacteria with antibiotic resistance whereas green tokens represent non-resistant ones.

Resistant and non-resistant populations of bacterial cells living into respectively the first and the second place at the microbiota level engage into a competitive battle whose dynamics are based on the interplay between their growth rates, availability of resources, and HGT mechanisms. The Petri-Nets model represents each of these mechanisms with a specific structure in the network architecture: HGT mechanisms are able to turn an existing non-resistant cell into a resistant one (Fig. 4g); both resistant and non-resistant species contribute to the overall population size, but through separate generative mechanism (Fig. 4d), which in this model, for simplicity, have the same growth rates; also the limited availability of resources bounds the population growth (Fig. 4e). From the Host level (Fig. 3) synchronous channels can be dynamically activated providing new inputs to the microbiota simulation: antibiotic administration events cause dose-dependent depletion of non-resistant cells (Fig. 4b) and microbiota reintegration causes a re-population into the state of non-resistance (Fig. 4f).

#### Bacterial cell level (bottom level)

In this general model, we did not implement the Bacterial Cells as separate Petri-Nets but simply as colored tokens. Tokens representing bacterial cells populate the two places at the Microbiota level in Fig. 4c. They are colored tokens, and their color is intended to carry information about species identity and the possible presence of resistance factors in the cell. They can be generated into the microbiota by either regular population growth mechanisms (Fig. 4d) or by non-resistant cells re-integration mechanisms (Fig. 4f). Obviously, individual bacteria could be modeled with a Petri-Net able to take into account much more complex dynamics like for example its gene regulatory network.

#### Antibiotic administration and spread of resistance (cross-layer communication)

All the three levels in the model are relevant for system representation, as well as the capability they have for exchanging information and affecting each other through dedicated channels.

Such exchanges occur dynamically along simulations, engaging the different levels in a continuous crosstalk, connecting them through selected channels. Allowing real-time selective communication is a requirement fulfilled by Renew [25].

For example, antibiotic administration events take place at the hosts (top) level (Fig. 3b) and affect all microbiota instances existing in the target place. In fact, the very effects of such events affect each single microbiota net-token, through the activation of the channel illustrated in Fig. 4b. Inside the single microbiota net-token, such events cause depletion of all tokens representing non-resistant cells, dramatically affecting the overall populations dynamics specific to that microbiota net instance.

Another communication mechanism involves the location of microbiota net-tokens at places reflecting their resistance status at the top level. Each place points to a condition: health, mild and severe resistance respectively. Moving a microbiota instance from a state to the next one requires the enabling and activation of the transition between the two (Fig. 3a). This transition is enabled only if a specific requirement is fulfilled: the PPS (dynamically computed at the microbiota level, in Fig. 4a) must cross the threshold corresponding to the establishment of the next resistance stage for the microbiota, that relocates it at the host level.

These two mechanisms involve exchanges of information among different model levels. In the first case, the host level imposes a way of functioning over the microbiota level, while in the second case it takes a decision according to information extracted from the microbiota level.

##### Model tuning

When dealing with multiple system levels, as often occurs in computational system biology [21], the model takes shape from different information and data sources. It is necessary to carry out careful data and model integration procedures so to leverage the available information. The capability of NWN models for hybridity allows for integrating existing models as long as consistency is guaranteed. This must be taken into account while choosing tuning the parameters of the model to obtain a good trade-off between knowledge representation and predictive capabilities.

In this context, we make a strong simplification: the large diversity of species composing a microbiota is recapitulated by tokens not carrying any information about their species, but only indicating whether they carry resistance capabilities or not. Nevertheless, the study of species diversity and how it is affected by antibiotic treatment (for an introduction to this problem, see [30]) is beyond the scopes of this model specification (but not out of the scope of the NWN formalism).

The proposed network architecture is based on a functional description of the problem at the different levels [31], while species identity and antibiotic or resistance mechanisms are maintained at an abstract level. The ultimate goal is to highlight some of the capabilities of a general instrument that can be easily adapted to the peculiarities of specific problems and use cases in the domain of interest.

#### Acinetobacter/E.coli model

The second model we present in this paper is very similar to the previous one at the Host level, but it is more detailed at the Microbiota level where it describes the dynamics of two common bacteria in the context of antibiotic resistance. This model is based on two experimental works treating the problem at the level of bacterial cells [27] and at that of microbiota hosts [28].

As introduced in the “Background” section, several bacterial species take part to the individual microbiota. Among them, Acinetobacterium acts as an opportunistic pathogen, posing a real threat to immunocompromised or injured individuals.

Besides the notoriety gained due to the threats it posed during recent military campaigns, Acinetobacterium Baumanii is known to be one of the most dangerous pathogens worldwide, having nosocomial mortality rates reaching 19-54% [26].

One of the reasons Acinetobacterium thrives in hostile environments such as hospitals or battle fields, is the capability to survive in difficult biotic and abiotic environments by setting up a protective biofilm. This causes the cells to stay in the environment longer, forming a pathogenic reservoir, and then increasing the probability of infection by environmental contact with other hosts.

As an additional cause for concern, this bacterial species seems to acquire resistance to antibiotics faster compared to many other bacterial species ([32, 33]). This causes Acinetobacterium to reach multi drug-resistance (MDR) rates beyond 60% ([26, 32]). As a possible explanation for this, in [27], authors show how a particular HGT mechanism, named *bacterial predation*, is able to increase cross-species HGT events by orders of magnitude. Bacterial predation involves a predator species (in this case Acinetobacterium) that, killing and destroying adjacent prey cells, acquires their adaptive resistance genes.

In [28], the authors study the same bacterial species at another system level: they focus on the spread and infectious activity of MDR Acinetobacterium strains in the gut microbiota of mice, with the scope of providing an in vivo model of post-surgery infections in hospitals. Also, they assess how a bacterial reintegration-based therapeutical approach is able to buffer and mitigate the rise of MDR Acinetobacterium, reducing then the severity of the infection and improving the health conditions of the infected host.

In this example we intend to model a system to study how Acinetobacterium special capabilities influence the overall resistance state of the microbiota and the prevalence of MDR. In this case, the three levels of the general model are customized to represent:

- the population of murine Hosts (top level)
- the mouse gut Microbiota (middle level)
- the Bacterial Cells (bottom level)

In the following sections, we provide a more detailed description of each level.

#### Population of murine hosts (top level)

At this level, the murine hosts receive the treatments indicated in the experimental design, i.e., antibiotic treatment possibly in combination with microbial reintegration. A dedicated network structure simulates the antibiotic administration (with the correct dosage and administration frequency) that is propagated to each net-token, which represents an individual microbiota. As in the previous model, each net-token can move among three possible places (no/mild/severe resistance) depending on its PPS score.

#### Murine gut microbiota (middle level)

**Fig. 6:**
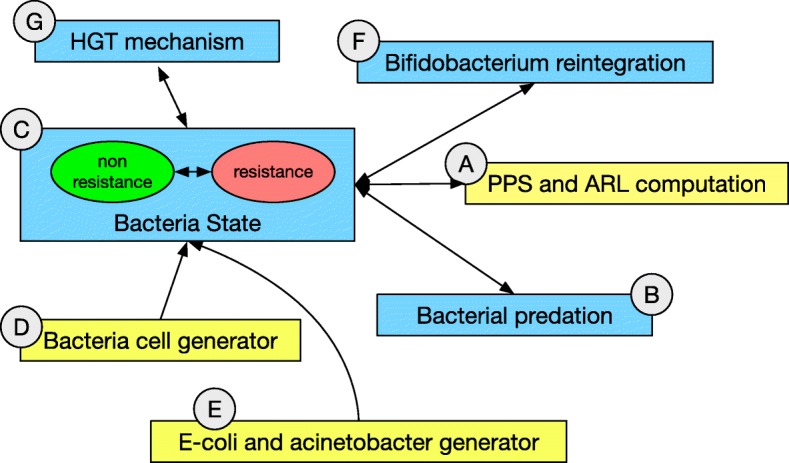
Conceptual view of the network architecture for the microbiotas (median) level in the Acinetobacterium/E.coli model

**Fig. 7:**
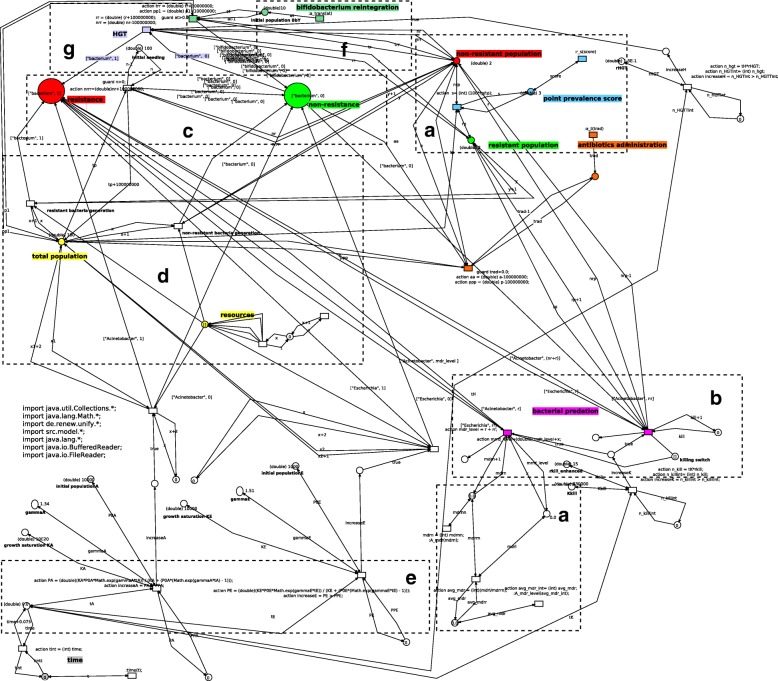
Network architecture for the microbiotas (median) level in the Acinetobacterium/E.coli model. Two main places describe two conditions each bacterial cell can assume: a state of non-resistance (green) and a state of resistance (red), respectively (**c**). Horizontal transfer mechanisms can turn non-resistant cells into resistant ones (**g**). Network structures managing the generation of new bacterial cells, including Acinetobacterium and E. Coli (**e**), total population numerosity and resources availability (**d**) give rise to a competitive population dynamics between resistant and non-resistant populations. Dose-dependent depletion of non-resistant cells following antibiotic administration (**b**) and Bifidobacterium reintegration (**f**) events activate as synchronous channels with structures in Fig. 3b. The two boxes marked with a highlight tracking structures in the model. One dynamically computes the point prevalence score (upper box) and the other (lower box) the Acinetobacterium Resistance Level of the microbiota network, making this information available for the upper level through synchronous channels

At this level we create a simplified model of the diversity inherent to the microbiota. Figure 6 shows its high-level structure while Fig. 7 reports its detailed implementation.

Different bacterial species compete for the ecological space in the gut niche. Development of MDR in Acinetobacterium cells emerges from standard HGT mechanisms (Fig. 7g) and from bacterial predation (Fig. 7b); both mechanisms are implemented with a dedicated structure. Predation can occur at variable rates [27].

Other bacterial species, from the point of view of Acinetobacterium studies, function also as preys and, possibly, as source of acquirable resistance factors.

At this level antibiotic resistance of the microbiota is quantified by its PPS score, and by the Acinetobacterium Resistance Level (ARL). The ARL is computed as the average amount of antibiotic resistant DNA factors acquired through bacterial predation divided by the number of total predation events. This score can be used to quantify the probability of a population to acquire multi-drug resistance factors.

#### Bacterial cells (bottom level)

Also in this second model bacterial cells are coded as colored tokens moving in the Microbiota level network. The color identifies the species (Acinetobacterium, E.Coli, Bifidobacterium, and generic commensal bacteria), and their antibiotic resistance level. Only tokens marked as Acinetobacterium can cumulate resistance factors through bacterial predation. In addition, an explicit modeling of time is provided to accurately integrate kinetic constants from [27] into the model. This allows us to compare the simulation outcomes with the experimental results, and to explicitly define timings for treatment administration or for emergence of particular environmental conditions relevant to the simulation (e.g., relative population densities favoring or not bacterial predation).

#### Parameter identification

The mechanism of bacterial predation involving Acinetobacterium and E.Coli is parametrized following [27] by adapting their mathematical descriptions to the network structures of this model. The most important parameters are reported in Table 1. Each simulation is initialized to reproduce the initial conditions presented in [28] to depict the untreated individuals. Quantitative information in the model refers to a single gram of microbiota. Killing enhancement described in [27] can reach a factor of 3 if the conditions in terms of relative population densities between predator and prey are optimal. In our model we set initial conditions so that the population sizes of Acinetobacterium and E.Coli favor the presence of the killing enhancement phenomenon.

**Table 1:** Model parameters

Model parameters	Values	Description
P0tot	10^10^	Total bacterial population
P0ByB	10^2^	Initial Bifidobacterium population
P0AB	10^2^	Initial Acinetobacterium population
KA	10^20^	Growth saturation for Acinetobacterium
KE	10^4^	Growth saturation for E. Coli
gammaA	1.34	Growth rate for Acinetobacterium
gammaE	1.51	Growth saturation for E. Coli
Killing rate (ehnanced)	150 h^-1^	Frequency of predation events
HGT rate	0.15 h^-1^	Frequency of horizontal gene transfer
		events

Quantities of bacterial cells are intended per gram of sample. Kinetic rates are intended per hour

## Results and discussion

To showcase the potential of multi-level and hybrid models for studying antibiotic resistance progression, we present three experimental designs for analyzing the problem focusing on different abstraction levels.

The first two experimental designs (ED1 and ED2) are based on the General Model. One experiment aims at evaluating how standard antibiotic treatments can cause and speed up the spread of resistance factors in a single microbiota. The second experiment is instead centered at the population level: multiple instances of the microbiota net are generated at the top level to represent a population of hosts distributed into the three defined resistance states and their dynamics is simulated. The third experimental design (ED3), executed on the Acinetobacterium/E.Coli Model, focuses on the effect of antibiotic treatments on the resistance level of a murine microbiota infected with Acinetobacterium.

In each experiment, the initial setting of the simulation represents hosts that did not receive any treatment administration, where the non-resistant bacterial population prevails largely over the resistant one.

Given the observed variance across simulations, we chose a number of 30 simulation runs per experimental condition, which guarantees a 5% error margin at a 95% confidence interval under all experimental designs.

### Experimental design ED1

**Fig. 8:**
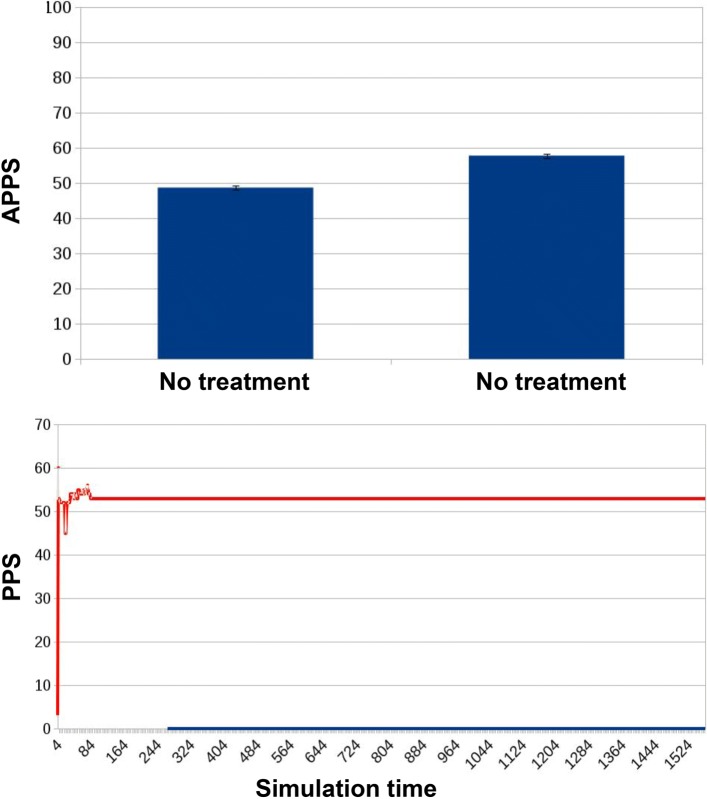
Results for ED1 in case of no treatment. The APPS is 48.67 ± 0.61 (first bar), under the threshold of mild resistance, keeping the microbiota into the “non-resistant” state. Only in 4 cases such threshold was crossed, and the APPS for those cases is slightly higher: 57.75 ± 0.63 (second bar). All cases had similar tracks, where PPS (the curve in orange) reaches a value and keeps it steadily along the simulation
- two administration events: a low dose followed by a second higher dose (Fig. 9);

In the first experimental design ED1, three different experimental conditions are considered:

- no antibiotic treatment (Fig. 8);

**Fig. 9:**
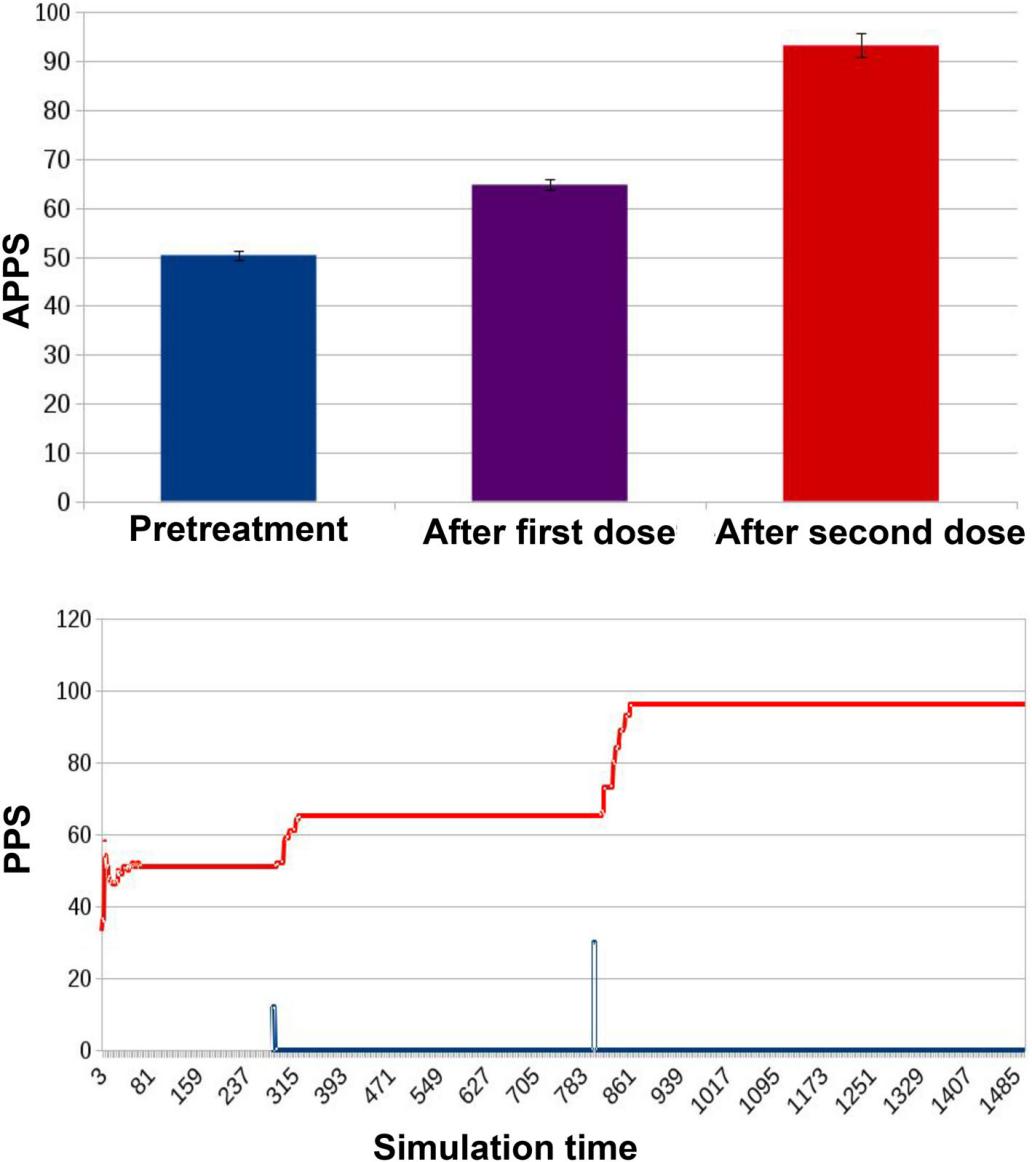
Results for ED1 in case of traditional treatment in which two doses of antibiotic were administered to the microbiota, the first lower and the second higher. The APPSs for the three significant stages of the experiment are: 50.34 ± 0.88 before treatment (homologous to that in Fig. 8), 64.74 ± 1.14 after the first dose (beyond the threshold for mild resistance) and 93.24 ± 2.4 after the higher dose, beyond the threshold for severe resistance. The simulation track of the PPS shows that after each dose (the curve in blue, whose peaks represent doses administration) PPS increases proportionally, moving from the “non-resistant” steady state, to the “mildly resistant” one, and finally to the “severely resistant” state, where it stays
- an innovative treatment protocol, where in parallel to the first low antibiotic dose, we simulate reintegration of non-resistant bacterial cells in the microbiota (Fig. 10). This reinforces the non-resistant population by mitigating the advantage acquired by the resistant cells after the antibiotic administration in the competition for colonizing the gut niche. This design is inspired by existing clinical practices such as autologous microbiota transplants, also called bacterial therapy [12].

**Fig. 10:**
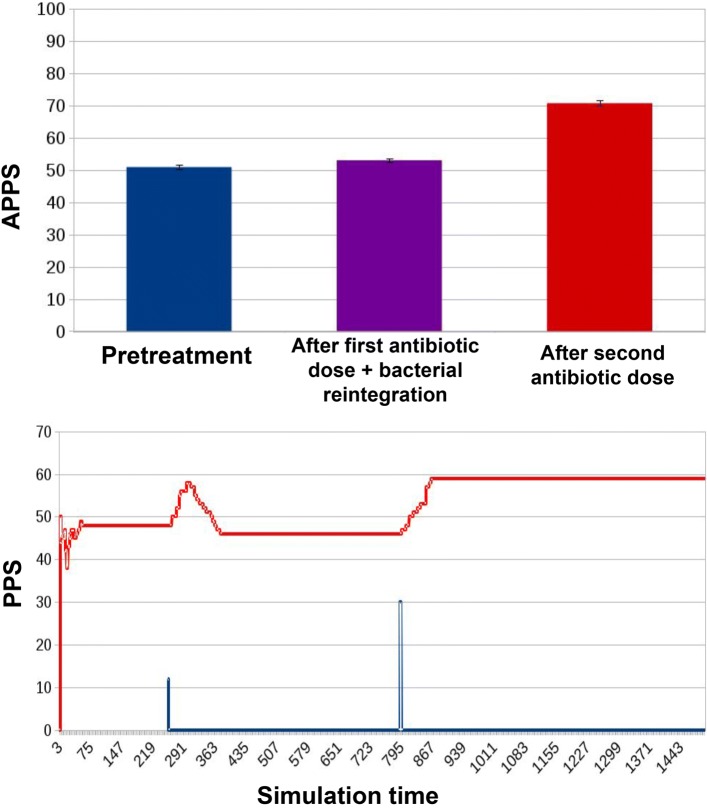
Results for ED1 in case of innovative protocol. We observe how this protocol lowers APPS both in the second and in the third stages of the experiment: while pretreatment APPS remains homologous to those in Figs. 8 and 9 (50.94 ± 0.67), after the first dose of antibiotic and microbiota reintegration it drops to 53.03 ± 0.52. Eventually, after the second dose of antibiotics it reaches 70.75 ± 0.82, corresponding to significant decreases compared to the corresponding simulation stages in the traditional treatment scenario Fig. 9. In the simulation curve, we observe how the effects of the first antibiotic dose on PPS are counterbalanced by the microbiota reintegration: in the first place PPS begins to rise, but it is bounded right away to a low level by the preventive action, leaving on the track just a transient spike. After the second, higher dosage of antibiotics, PPS increases, reaching a steady state at a higher level, which is anyway lower that that reached in Fig. 9

In this first experimental design, simulations track the PPS of a single microbiota; this measure is correlated with the presence of resistant cells within the total bacterial population. PPS score is sampled at different stages of the simulation:

- before the administration of any treatment;
- after the administration of the first, low dose of antibiotic (plus, for the innovative protocol, the microbiota integration)
- after the administration of the second higher dose of antibiotics.

The values presented in Figs. 8, 9, 10 are computed as the average point prevalence score (APPS) over 30 simulation runs. Each simulation is slightly different thanks to the stochasticity introduced in the model. This type of simulation allows us to study, given the internal population dynamics of resistant and non-resistant species, the link between the overall resistance state of a microbiota and the different treatment protocols.

In the first experimental condition, when no treatment is provided, the APPS remains at a low and almost constant level during the whole simulation. Small increases are caused by the activation of random mutations and/or horizontal gene transfer events. In only 4 out of 30 experiments the score exceeded the threshold required to assign the microbiota into a state of mild resistance. The relative averaged APPS of 57.75 ± 0.63 (second bar of Fig. 8) refers to these four cases. All remaining simulations are represented by the first bar of Fig. 8, showing that the microbiota remained in an healthy state, with an APPS score of 48.67 ± 0.61. In none of the simulations the microbiota reached a state of severe resistance.

In the second experimental condition, the model simulates a treatment protocol composed of two administrations of an increasing dose of antibiotic (Fig. 9). Before treatment (as in the control condition), the APPS was 50.34 ± 0.88. The administration of the first lower dose allows a partial recovery of the non-resistant portion of the bacterial population (APPS of 64.74 ± 1.14); the second higher dose, instead, takes the microbiota towards a state of increased resistance (APPS 93.24 ± 2.4).

In the third experimental condition (Fig. 10), the previous treatment protocol is improved with a parallel preventive reintegration of non resistant bacterial cells; this improved protocol has a significant effect after each antibiotic dose: the APPS decreases of 18.08% after the first dose (when compared to that originating from the traditional treatment alone), and decreases of 24.12% w.r.t. the traditional protocol after the second antibiotic dose.

If we look at the PPS during the whole simulation, it is evident how it is constant when no treatment is administered (Fig. 8). With a traditional treatment, it increases in correspondence with the administration of the two antibiotic doses (blue curve in Fig. 9). The same dynamic can be observed in Fig. 10, with the difference that the preventive action of bacterial reintegration mitigates the resistance effects of the first dose of antibiotics, taking the PPS score to a level similar to pre-treatment.

### Experimental design ED2

**Fig. 11:**
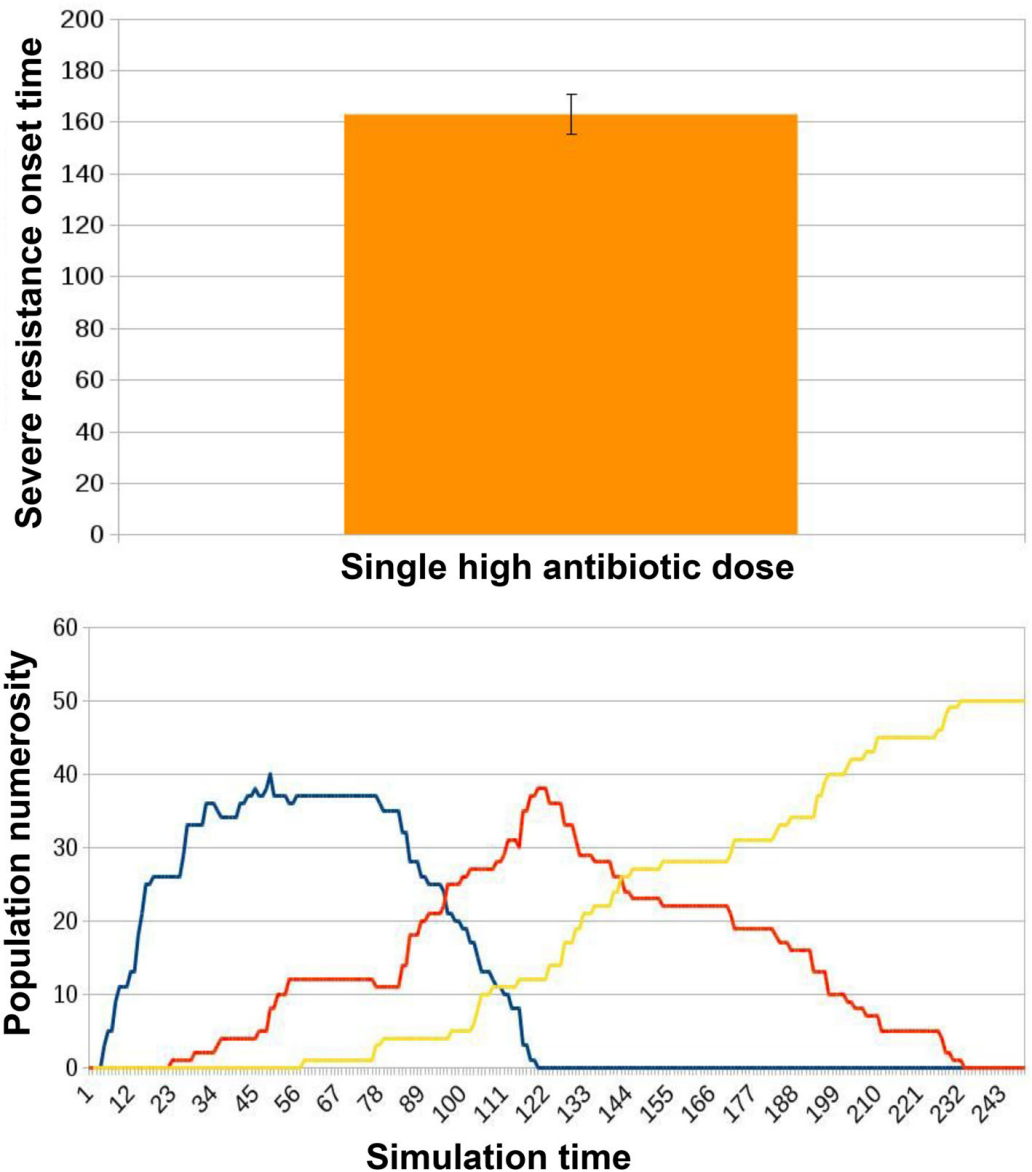
Results for ED2 in case of traditional treatment in which a single high dose of antibiotic is administered to the microbiota. The administration of a single high dose of antibiotics was presented to each microbiota instance. This corresponded to an average onset time for severe resistance (AT) of 162.87 ± 7.78. Simulation tracks from these experiments show healthy individuals (blue curve) progressively acquiring resistance. Some of them reach severe resistance right away (yellow curve), while most of them pass through a phase of mild resistance (orange curve)
- a preventive bacterial reintegration occurs in parallel to the single high-dosage antibiotic administration (Fig. 12).

The second experimental design ED2 focuses on the host population level; we created multiple instances of the microbiota net-token in order to represent a population of hosts. In this second experiment, we simulated the following experimental conditions:

- in the control condition no treatment is administered;
- a single, high-dosage administration event takes place (Fig. 11);

**Fig. 12:**
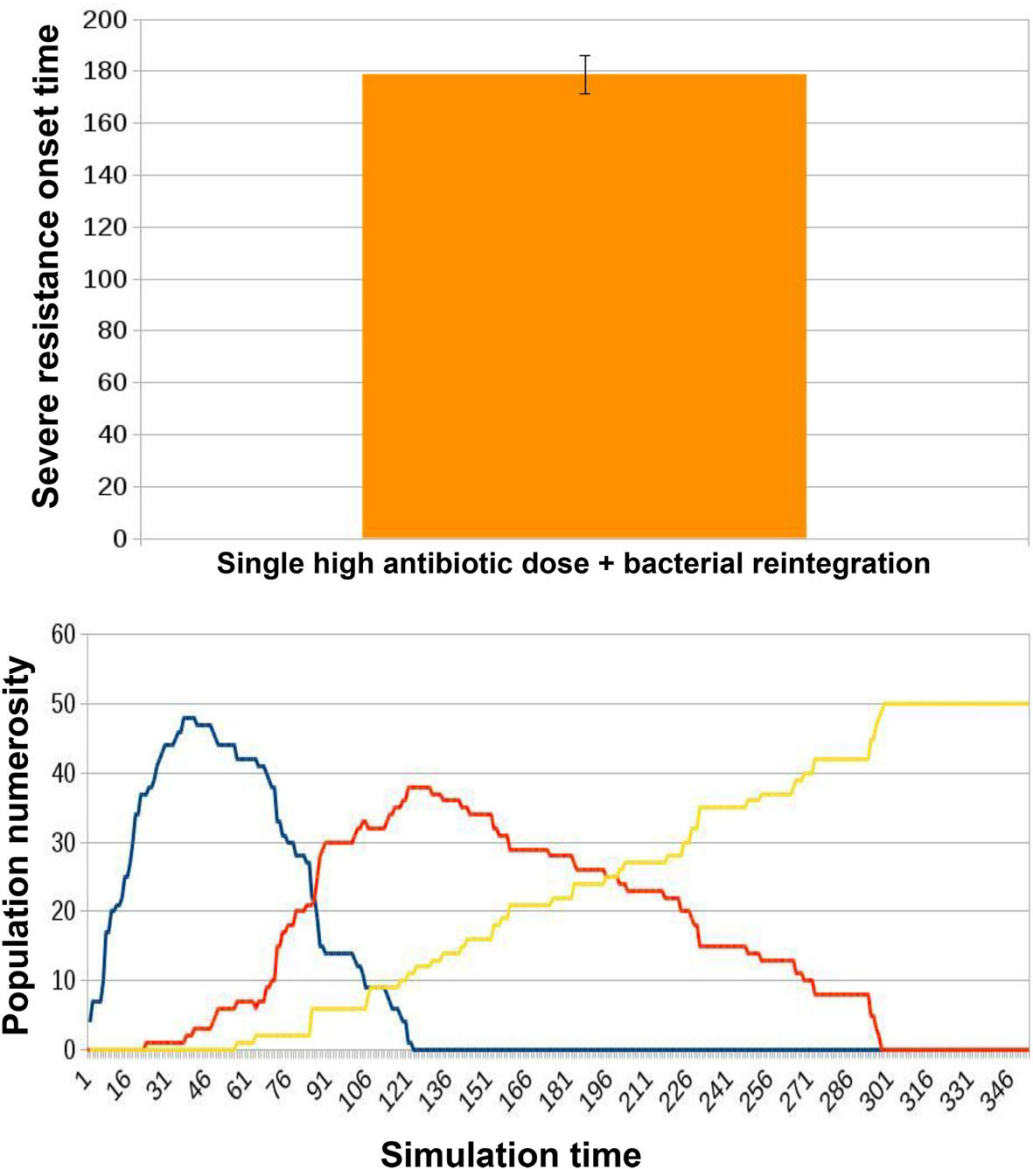
Results for ED2 in case of innovative treatment in which a single high dose of antibiotic combined with bacterial reintegration is administered to the microbiota. Microbiota reintegration combined with antibiotic administration yields an increased AT (178.64 ± 7.4) with respect to that observed with the administration of the antibiotics alone Fig. 11. In simulation tracks we notice how the overall migration of healthy individuals towards a worsening resistant state slows down, resulting in a slower severe resistance onset time (T)

We simulated 50 instances of the microbiota network. As a metric to analyze the evolution of the population we consider the simulation time at which the number of hosts in a state of severe resistance exceeds the number of hosts in a state of mild resistance (that we define as the severe resistance onset time, T); this represents a landmark of the process degeneration towards extensive resistance spread and diffusion.

When no treatment is administered, microbiotas do not get to a state of severe resistance. Instead, as we can observe in Fig. 11, with a single high-dosage administration of antibiotic alone, the onset of a state of prevalent severe resistance within the hosts population is observed at an average onset time (AT) of 162.87 ± 7.78. When microbiota re-integration is provided in parallel with the antibiotic dose, there is an average delay of 9.68% in AT, 178.64 ± 7.4, see Fig. 12. Examples of the typical temporal dynamics in the two experimental conditions are provided respectively in Figs. 11 and 12.

### Experimental design ED3

In this last experimental design, we compare the behavior of an untreated microbiota, another one receiving antibiotic treatment, and finally a third one receiving antibiotic treatment combined with bacterial reintegration. The objective is to observe the spread of resistance levels in the Acinetobacteria and overall microbiota population.

The experiments are organized on the following timeline:

- at day 0 (in [28], day -7) daily administration begins and it goes on until day 14.
- at day 7 (in [28], day 0: infection with MDR Acinetobacterium relative bacterial population densities arise so to cause a killing enhancement of a factor of 2 in Acinetobacteria, corresponding to increasing the probability that they acquire MDR.
- at day 14 the experiment is interrupted, and the variables of interest assessed.

Simulation of treatment includes antibiotic administration and, possibly, bacterial reintegration with Bifidobacterium Fig. 7f.

Simulations cover the time period of 14 days, and time is explicitly modeled and simulated. To assess how the different modelled mechanisms affect the spread of antibiotic resistance, we track both PPS scores and, only for Acinetobacteria, ARL scores at the end of the first, the seventh, and the fourteenth days in the simulation timeline. Time intervals replicate those from the experimental design of [28].

**Fig. 13:**
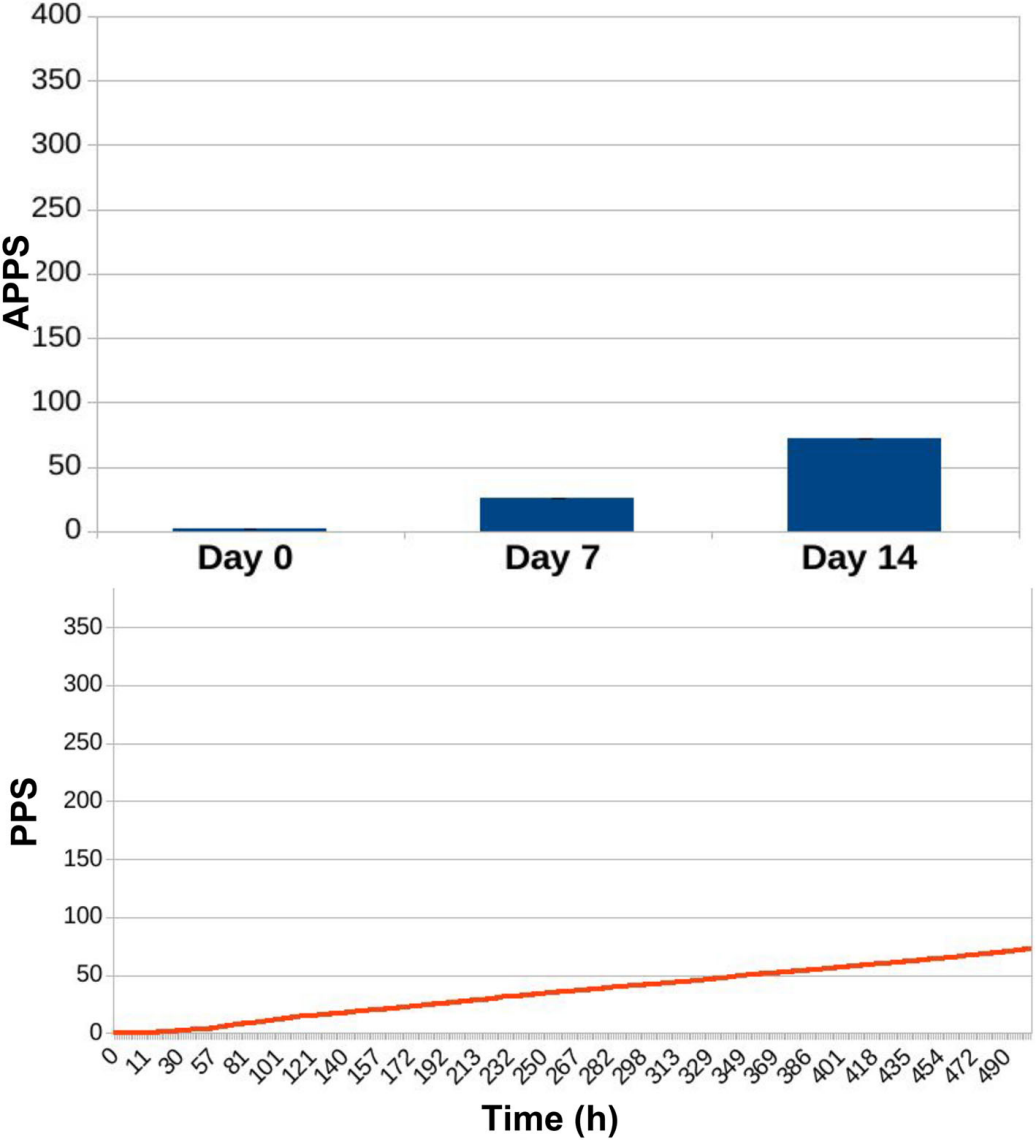
Results of PPS tracking for ED3 in case of no treatment administration to the murine microbiotas. When no treatment was administered to murine hosts, AP is 2.27 ± 0.04 at day 0, 25.3 ± 0.07 at day 7 and reaches 72.29 ± 0.07 at the end of the experiment, at day 14. Simulation tracks show PPS increasing steadily and slowly compared to Fig. 14)

**Fig. 14:**
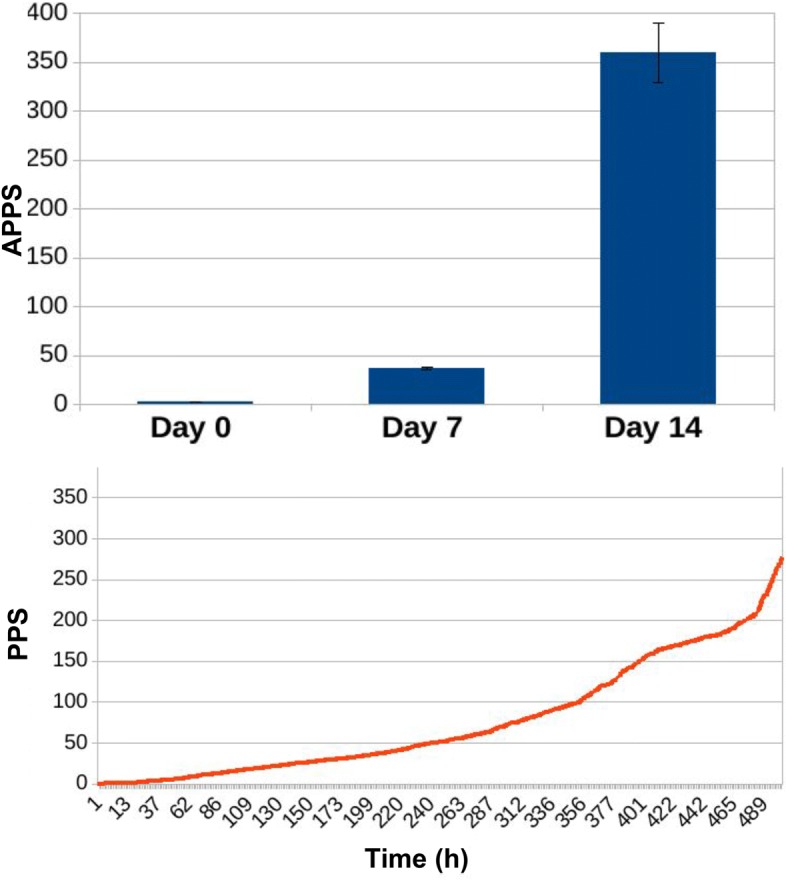
Results of PPS tracking for ED3 in case of daily administration of antibiotic treatment to the murine microbiotas. Simulating daily administration of antibiotic doses to the mice we observe, for the three relevant time points of the experiment, an AP of 2.5 ± 0.05 (day 0), very close to the result in Fig. 13; after seven days of daily antibiotic administration, AP is 37.57 ± 1.04 (day 7), and after seven days more reaches the value of 359.21 ± 30.11 (day 14). The simulation curve shows how PPS grows faster compared to Fig. 13, reflecting the movement of population dynamics towards the establishment of the domination of the microbiota by resistant species

**Fig. 15:**
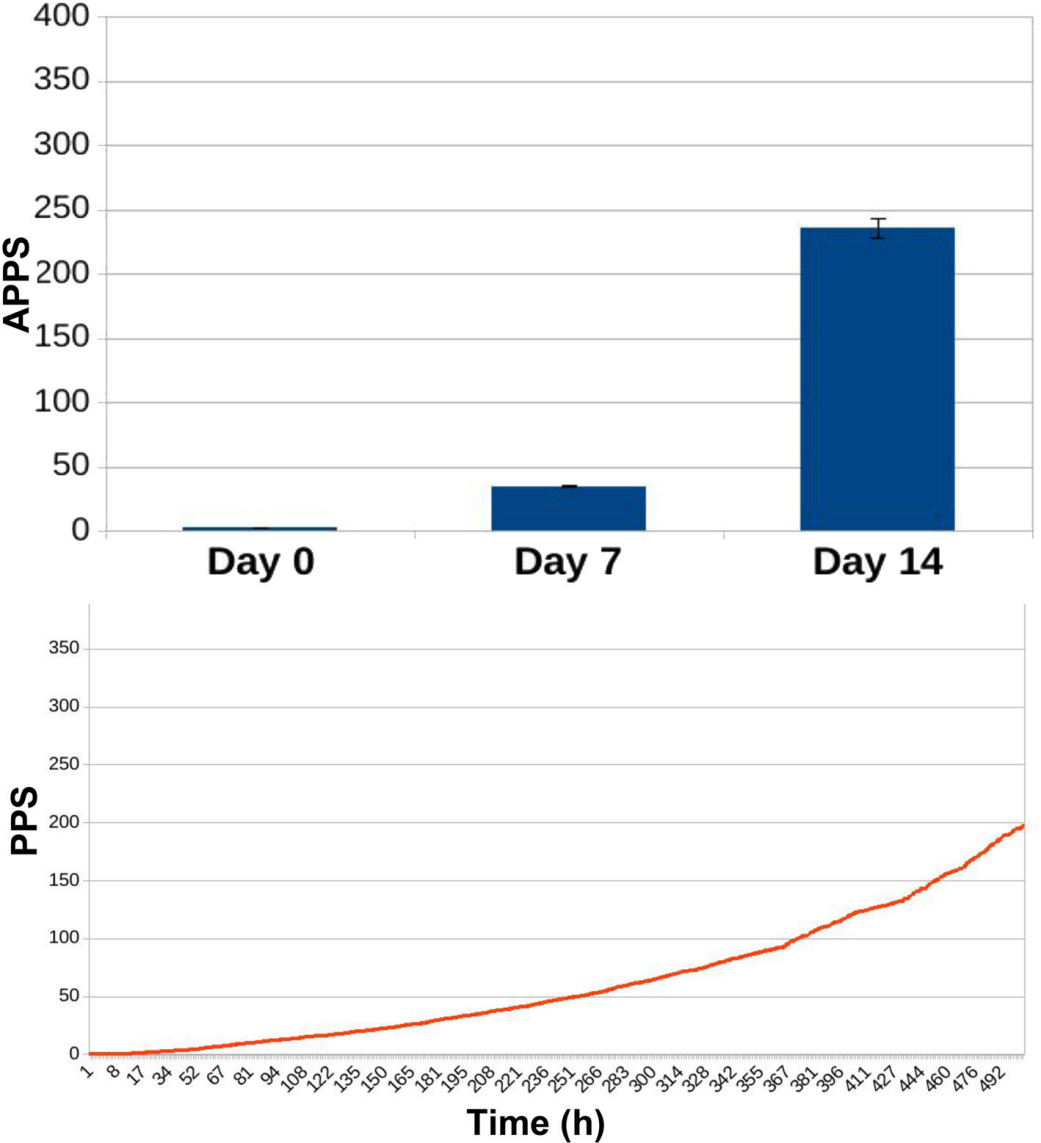
Results of PPS tracking for ED3 in case of daily administration of antibiotic treatment combined with Bifidobacterium reintegration to the murine microbiotas. The combination of antibiotic administration and bacterial reintegration lowers AP values both at day 7 and 14. While AP at day 0 has a value of 2.54 ± 0.04, similar to those reported in Figs. 13 and 14, after seven days of combined treatment daily administration AP is 35.25 ± 0.51, and at day 14 reaches the value of 235.94 ± 7.9. This assesses the mitigating action by bacterial reintegration over the resistance levels observed when simulating the administration of antibiotic treatment alone Fig. 14. Simulation tracks show how PPS grows in time at a pace slower than that observed in case of antibiotic treatment alone(Fig. 13)

**Fig. 16:**
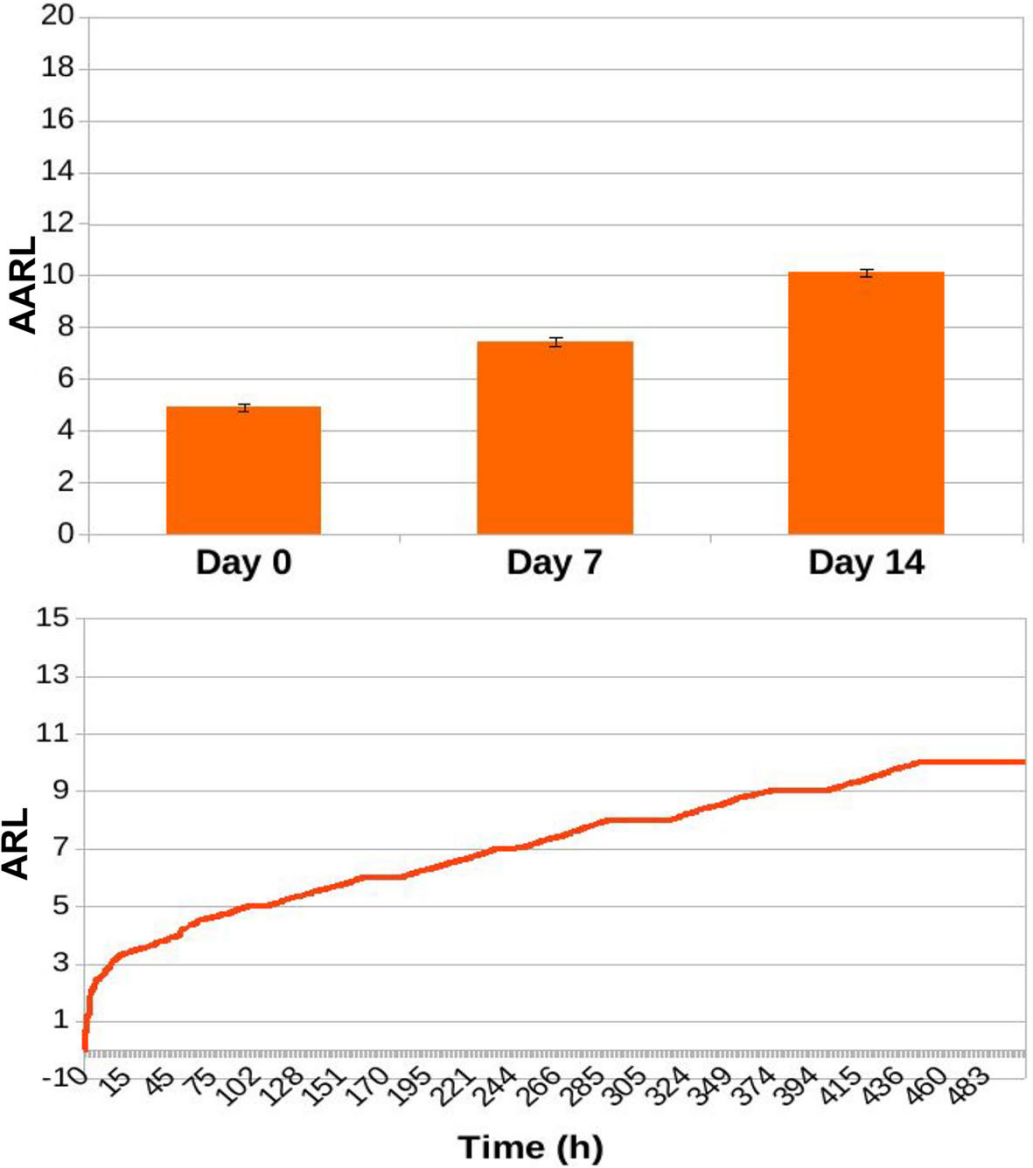
Results of ARL tracking for ED3 in case of no treatment administration to the murine microbiotas. When simulating a condition of absence of treatment, average ARL (AARL) assumes values of 4.83 ± 0.12 at day 0, 7.6 ± 0.19 at day 7 and 10.33 ± 0.18 at the end of the experiment, at day 14. Simulation tracks show a slow and steady increase of ARL along simulation time

**Fig. 17:**
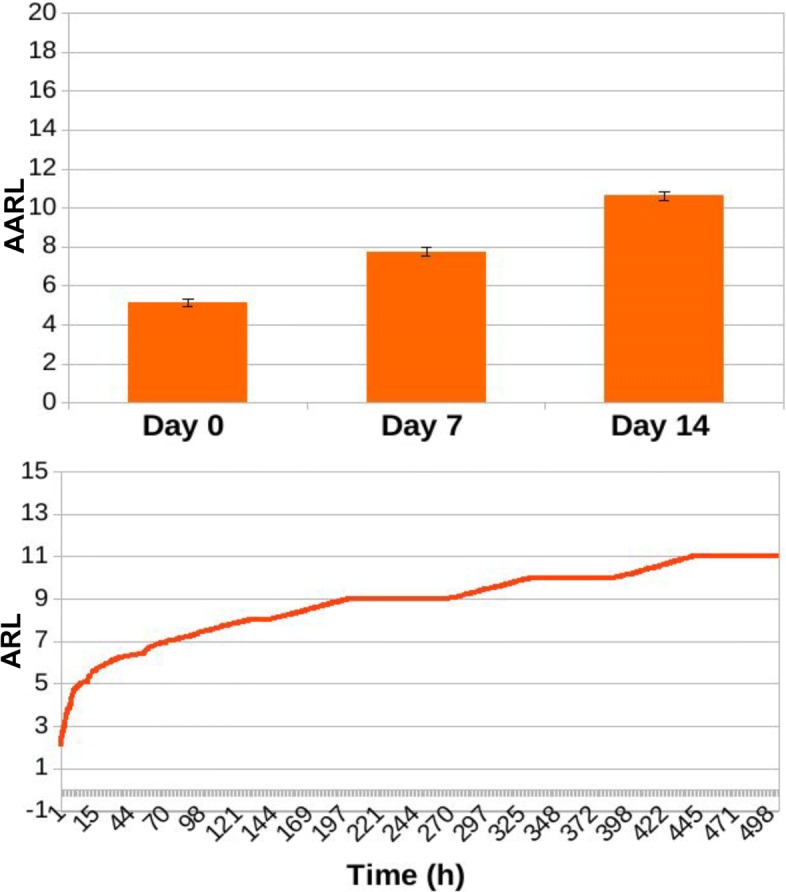
Results of ARL tracking for ED3 in case of daily administration of antibiotic treatment to the murine microbiotas. Simulation of daily antibiotics administration to the murine microbiotas yields to AARL values of 5.12 ± 0.2 at day 0, 7.77 ± 0.23 at day 7 and 10.61 ± 0.22 at day 14. Similarly to Fig. 16, simulation tracks show a slow and steady increase of ARL along simulation time

**Fig. 18:**
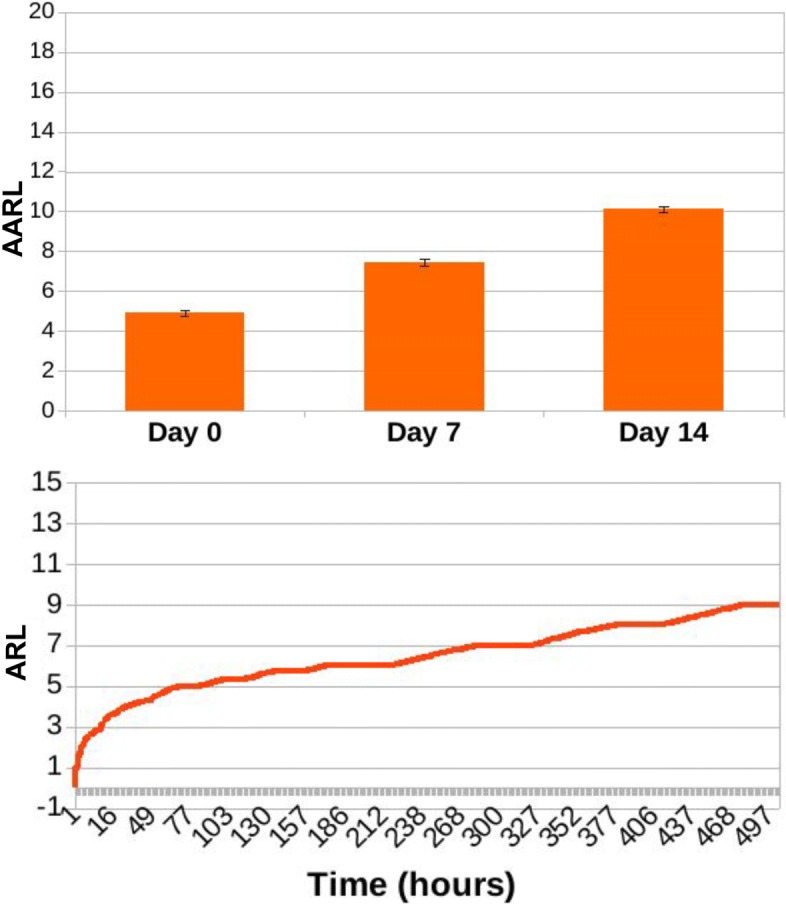
Results of ARL tracking for ED3 in case of daily administration of antibiotic treatment combined with Bifidobacterium reintegration to the murine microbiotas. Combining Bifidobacterium reintegration to antibiotic doses in daily treatment administration AARL is 4.93 ± 0.14 at day 0, 7.46 ± 0.16 at day 7, and 10.13 ± 0.15 at day 14, and simulation tracks show a slow and steady increase of ARL along simulation time, similarly to what it is observed both in case of no treatment administration (Fig. 16) and of daily antibiotic administration alone (Fig. 17)

In Figs. 13, 14, 15, 16, 17, and 18 we separately present results for PPS and ARL tracking respectively.

Antibiotic administration causes average PPS to increase significantly, especially at day 14: while in the first experimental condition (no treatment) APPS is 72.29 ± 0.07 (Fig. 13), in the simulations including antibiotic treatment it reaches the value of 359.21 ± 30.11 Fig. 14. In Fig. 15 we observe how the combination of antibiotic administration and bacterial reintegration lowers the score both at day 7 and 14 compared to the case where antibiotics alone were administered; after seven days of the combined treatment APPS is 35.25 ± 0.51, and at day 14 it reaches the value of 235.94 ± 7.9.

As it can be observed, average ARL (AARL) tends to increase along simulation time, reflecting the growing pool of DNA exchanged through predation by Acinetobacterium. No significant variations are observed across experimental conditions (Figs. 16, 17, 18), reporting that the MDR level in Acinetobacteria grows steadily during the time of the experiments, as exemplified by the simulation tracks.

These results considered together are coherent with those presented in [28], where the effect of bacterial reintegration is measured evaluating the spread of resistant Acinetobacterium infection in the host during antibiotic treatment. They show reductions of the Acinetobacterium infection spread when bacterial reintegration combines with antibiotic treatment compared with the case antibiotic treatment is administered alone. Considering that as an indirect measure for the resistance level in Acinetobacteria, our results, indicating a 30% reduction of resistance level in the whole microbiota in case of bacterial reintegration, assess the capability of our model for making consistent predictions.

Even though antibiotic treatment and bacterial reintegration do not seem to affect the propensity of Acinetobacterium towards acquiring exogenous, potentially resistant DNA (as shown in Figs. 16, 17, 18), they do affect the overall resistance level of the microbiota, and thus the probability a predation event leading to the acquirement of multi-drug resistance.

## Conclusions

In this work, we suggest some potential applications of NWN models for investigating the relations between antibiotic treatment and the spread of antibiotic resistance within a microbiota, and across hosts populations. The use of a multi-level and hybrid formalism allows integrating different types of data, as well as to describe the system under study at multiple levels: in the context of antibiotic resistance, they allow modeling the system from the level of bacterial cells and resistance-carrying molecules, to the microbiota they are part of, up to the population of hosts undergoing different treatments and interacting between each other. Simulations enable to take into account the timing and stochastic behaviors required to model administration protocols as well as the stochasticity intrinsic to biological processes.

In this work we want to underline how models of this kind not only provide valuable tools for investigating causal, quantitative relations between different events and mechanisms, but can be used as supports for decision making processes and clinical protocols development.

The presented models, in their current form, present strong simplifications. Nevertheless, their flexibility makes it easy to adapt them to specific and more realistic and detailed use cases, built gathering realistic data from the field.

Our future work includes applying the presented modeling strategy to first-hand data. This will be the first step towards the combination of hypothesis-driven model specification with data-driven parameter identification. This combination, along with the consistent model and information integration processes, will allow to extract new knowledge, to guide new experiments, and to generate new data from the systems of interest.
